# U-shaped association between serum 25-hydroxyvitamin D concentrations and urinary leakage among adult females aged 45 years and over in the United States: a cross-sectional study

**DOI:** 10.1186/s12905-024-02906-6

**Published:** 2024-01-23

**Authors:** Zeyu Li, Xinzhuo Lu, Keshuai Zhang, Shuangyan Wu, Wei Yu, Xiaoling Chen, Wenzhong Zheng

**Affiliations:** 1 Department of Urology, Fujian Medical University Union Hospital, 29 Xinquan Road, Gulou District, Fuzhou, 200001 Fujian Province P.R. China; 2 School of Basic Medical Sciences, Fujian Medical University, Fuzhou, China; 3 Department of Intensive Care Unit, Fujian Medical University Union Hospital, Fuzhou, China

**Keywords:** Serum 25-hydroxyvitamin D, Urinary leakage (UL), National Health and Nutrition Examination Survey (NHANES), US Middle-aged females, U-shaped association

## Abstract

**Background:**

The relationship between serum vitamin D status and urinary leakage (UL) among middle-aged females needs to be further studied. The aim of this study was to evaluate the association of serum 25-hydroxyvitamin D [25(OH)D] concentrations with UL among American females ages 45 years and over.

**Methods:**

Seven cycles of the National Health and Nutrition Examination Survey (NHANES) with self-report UL data, were used. A total of 9525 women aged 45 years and older were enrolled in this study. Univariate and multivariate logistic regression models and the smooth curve fitting were utilized to analyze the association between clinical UL and serum 25-hydroxyvitamin D [25(OH)D] concentrations.

**Results:**

A non-linear relationship between serum 25(OH)D concentrations and clinical ULwas observed. When serum 25(OH)D concentration was higher than the inflection point 63.5 nmol/L, a positive correlation was observed between serum 25(OH)D concentrations and clinical UL ([OR]: 1.007, 95%CI: 1.005–1.009, *P* < 0.01). However, when serum 25(OH)D concentration was below the inflection point 63.5 nmol/L, a negative correlation was observed between serum 25(OH)D concentrations and clinical UL ([OR]: 0.993, 95%CI: 0.989–0.996, *P* < 0.01).

**Conclusions:**

The association between serum vitamin D and the risk of UL exhibited a U-shaped pattern among US middle-aged females, with an inflection point occurring at a serum 25(OH)D concentration of 63.5 nmol/L.

## Introduction

Urinary leakage (UL) or urinary incontinence (UI) is a common and often inadequate treated condition that affects millions of American women [1], and the prevalence increases with age and vaginal delivery [2]. In a research population followed for 8 years, an overall incidence rate of UL of 21.9% (95% CI, 19.6-24.2%) and a remission rate of 33.3% (30.1‐36.5%) were observed [3]. UL can be caused by various factors such as age-related physiologic changes, urological or gynecological diseases, neurological illnesses, behavior patterns and functional decline frequently [4]. It affects personal health, reduces the quality of life, and increases the economic burden of individuals and society.

25-hydroxyvitamin D [25 (OH) D], the main stored form of serum 25(OH)D, is a fat-soluble substance that promotes calcium and phosphate absorption [5]. Serum 25(OH) D deficiency is a significant risk factor for various conditions, including myasthenia gravis [6], type 2 diabetes [7], chronic kidney disease [8], cardiovascular disease [9], and asthma [10]. However, low levels of serum 25(OH)D which defined as serum 25-hydroxyvitamin D below 30 ng/ml influences more than two thirds of the U.S. adult population and approximately one billion individuals in the whole world [11, 12]. Numerous studies have found that serum 25(OH)D is associated with various urological diseases, such as hyperuricemia [13], male lower urinary tract symptoms and benign prostatic hyperplasia [14], and urological cancer [15]. Furthermore, multiple clinical trials have examined the negative impact of serum 25(OH) D deficiency on pelvic floor muscle strength, an essential components of pelvic floor support [16, 17].

Although previous studies have demonstrated that lower serum 25(OH)D concentrations were associated with UL [18, 19], the current evidence proving the relationship between serum 25(OH)D status and UL is limited and inconsistent. For example, a cross-sectional study found a high prevalence of serum 25(OH)D deficiency in stress UI patients [19], while another study indicated that the risks of developing UL were not related to vitamin D intake categories in middle-aged and older women [20].

Numerous studies have been conducted to investigate the relationship between UL and serum 25-hydroxyvitamin D levels. However, the specific scenarios in which this link has been studied remain limitedand and inconsistent. To address these confusions, based on data from the National Health and Nutrition Examination Survey (NHANES), this study was carried out to investigate the association of serum 25(OH)D concentrations with risk of UL among U.S. women age 45 and older.

## Materials and methods

### Study population

**Fig. 1:**
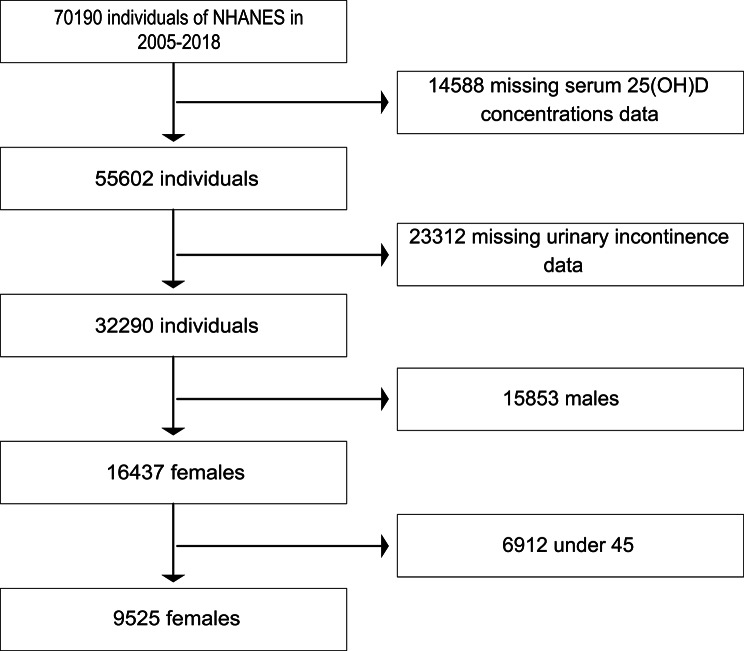
Flow chart of study participants. Seven cycles of NHANES data including 70,190 individuals from 2005 ~ 2018 were primary screened. 14,588 volunteers with missing serum 25(OH)D concentrations data and 23,312 individuals with missing urinary leakage data were dropped. In addition,15,853 males and 6912 volunteers under the age 45 were excluded. Finally, 9525 females were included in this study

The National Health and Nutrition Examination Survey (NHANES) is performed by the National Center for Health Statistics (NCHS) of the Centers for Disease Control and Prevention (CDC), consisting of approximately 10,000 persons every two years. The purpose of the survey is to generate national estimates that accurately represent the general non-institutionalized civilian population of the United States. It collected information on demographic data and health condition through interviews, physical examinations and laboratory tests. The institutional review board of the NCHS approved the NHANES study protocols and released the data while protecting the privacy of the participants. And all participants submitted written informed consent. In this study, we utilized these data strictly for analysis and reporting purposes. The NHANES data can be accessed through the official NHANES website [21]. In this study, we conducted a secondary analysis using data from the seven cycles of NHANES with self-report UL (2005–2006, 2007–2008, 2009–2010, 2011–2012, 2013–2014, 2015–2016, and 2017–2018). Given the high incidence of UL in middle-aged and older women, our study focused on female participants over the age of 45 years old with complete UL and vitamin D data. The flowchart of participant selection was demonstrated in Fig. 1.

### Variables

In the NHANES 2005–2006, serum 25(OH)D concentrations were determined using DiaSorin radioimmunoassay kit (Stillwater, MN). Starting from the 2007 to 2008 cycle, serum 25(OH)D concentrations were determined using a standardized liquid chromatography-tandem mass spectrometry (LC-MS/MS) method. Serum 25(OH)D data from NHANES 2005–2006 were converted to equivalent 25(OH)D measurements using the LC-MS/MS method by regression equations. Additional information can be found on the NHANES website [22]. In accordance with CDC recommendations, the LC-MS/MS-equivalent data was used [22].

In addition, Some covariates from the NHANES database, including age, race (White and Nonwhite), education, income, drink, smoke, BMI, diabetes, hypertension, hyperlipidemia, stroke, asthma, hysterectomy, pregnant and vaginal deliveries were used. UL was accessed in NHANES through a question, how often have urinary leakage (Question number: KIQ005). Participants who responded “every day and/or night”, “a few times a week”, “a few times a month” or “less than once a month” to this question were classified as UL.

### Statistical analysis

The data in this study were analyzed statistically in accordance with CDC guidelines. Serum 25(OH)D levels were modeled and analyzed as continuous and categorical variables. Serum 25(OH)D concentrations were categorized into four groups according to the Endocrine Society Clinical Practice Guidelines, as follows: severe deficiency (< 25.00 nmol/L), moderate deficiency (25.00-49.99 nmol/L), insufficient (50.00-74.9-9 nmol/L), and sufficient (≥ 75.0 0nmol/L). The selection of appropriate sample weights for the analysis depends on the variables being used. All interview and MEC (Mobile Examination Center) exam weights covered in this study are available in the demographic files. We utilized mean and standard deviation to summarize continuous variables, while frequency and percentage were used for categorical variables. To compare whether there were any statistical differences between different vitamin D groups, a weighted linear regression model was used for continuous variables and the weighted chi-square test for categorical variables. The relationship between UL and serum 25(OH)D levels was evaluated using univariate and multivariate linear regression models as follows: Unadjusted model: No adjustment; Adjusted model: Adjusted for age, race, education, income, BMI, drink, smoke, diabetes, hypertension, hyperlipidemia, pregnant and vaginal deliveries history. Multiple imputation was performed for covariates with missing values. The nonlinear relationship between serum 25(OH)D levels and UL was determined using smooth curve fitting and a generalized additive model. The inflection point with the maximum model likelihood value was determined using the trial-and-error method, and the log-likelihood ratio test was performed for the one-line linear model and the two-piecewise linear model. All analyses were performed using software package R (http://www.R-project.org, The R Foundation). In this study, a two-sided *P*-value of less than 0.05 was considered statistically significant.

## Results

### Baseline characteristics

Between 2005 and 2018, a total of 9525 participants were finally enrolled in our analysis. The characteristic distribution of participants was presented in Table 1 (according to serum 25(OH)D concentrations), Table 2 (according to UL) and Table 3 (according to severity of UL). Briefly, women with higher levels of serum 25(OH)D tended to be older, white race, have a lower BMI, higher levels of education and higher incomes (*p* < 0.01). Interestingly, serum 25(OH)D concentrations were found to exhibit a non-linear relationship with several variables including hypertension, stroke, and UL (Table 1). According to UL, females in UL group had higher serum 25(OH)D levels (*p* < 0.01) (Table 2). Furthermore, patients in the severe UL group had significantly higher serum 25(OH)D levels compared to those in the mild UL group (*p* < 0.01) (Table 3).

**Table 1:** Baseline characteristics of participants with UL according to serum 25(OH)D concentrations

Characteristic	Serum 25(OH)D concentrations (nmol/L)				***P*** value	***P*** for trend
	< 25.00 (*n* = 353)	25.00–49.99 (*n* = 2204)	50.00–74.99 (*n* = 3084)	≥ 75.00 (*n* = 3884)		
**Age (mean (SD))** ^$^	60.44 (10.13)	60.40 (10.66)	61.05 (10.71)	64.85 (10.83)	< 0.001	< 0.001
Race_2 = White (%)*	62 (17.6)	634 (28.8)	1309 (42.4)	2370 (61.0)	< 0.001	< 0.001
Education (%)*					< 0.001	< 0.001
*College or above* ^$^	125 (37.2)	839 (39.4)	1291 (43.2)	1967 (52.3)		
*High school or equivalent*	175 (52.1)	947 (44.4)	1266 (42.4)	1446 (38.4)		
*Less than high school*	36 (10.7)	346 (16.2)	430 (14.4)	350 ( 9.3)		
**Income (mean (SD))** ^$^	2.01 (1.39)	2.26 (1.54)	2.49 (1.59)	2.88 (1.63)	< 0.001	< 0.001
Drink = Yes (%)*	112 (57.4)	684 (55.8)	939 (56.8)	1259 (61.8)	0.002	0.005
Smoke = Yes (%)*	162 (45.9)	909 (41.3)	1143 (37.1)	1478 (38.1)	< 0.001	0.016
**BMI (mean (SD))** ^$^	33.60 (9.12)	32.12 (7.79)	30.17 (7.02)	28.65 (6.84)	< 0.001	< 0.001
Diabetes = Yes (%)*	94 (26.7)	496 (22.5)	551 (17.9)	657 (16.9)	< 0.001	< 0.001
Hypertension = Yes (%)*	211 (59.9)	1214 (55.3)	1535 (49.8)	2162 (55.7)	< 0.001	< 0.001
Hyperlipidemia = Yes (%)*	150 (46.9)	910 (45.3)	1437 (50.4)	2028 (53.8)	< 0.001	0.001
Stroke = Yes (%)*	33 ( 9.3)	134 ( 6.1)	165 ( 5.4)	247 ( 6.4)	0.019	0.019
Asthma = Yes (%)*	68 (19.3)	348 (15.8)	483 (15.7)	615 (15.8)	0.36	0.478
Hysterectomy = Yes (%)*	127 (36.2)	748 (34.8)	1040 (34.5)	1485 (38.7)	0.001	0.115
Ever_pregnant_level (%)*					< 0.001	< 0.001
*1 ~ 2*	111 (33.9)	596 (29.7)	846 (30.2)	1260 (36.0)		
*3 ~ 5*	157 (48.0)	1068 (53.1)	1531 (54.6)	1825 (52.1)		
*> 5*	59 (18.0)	346 (17.2)	426 (15.2)	415 (11.9)		
Vaginal_deliveries_level (%)*					< 0.001	0.004
*0*	52 (15.8)	309 (15.2)	374 (13.2)	462 (13.1)		
*1 ~ 2*	121 (36.8)	711 (35.0)	1085 (38.4)	1492 (42.4)		
*3 ~ 5*	131 (39.8)	825 (40.7)	1136 (40.2)	1346 (38.3)		
*> 5*	25 ( 7.6)	184 ( 9.1)	231 ( 8.2)	216 ( 6.1)		
UL_bin = Yes (%)*	191 (54.1)	1095 (49.7)	1530 (49.6)	2144 (55.2)	< 0.001	0.082
UL_level (%)*					< 0.001	0.144
*Never*	162 (45.9)	1109 (50.3)	1554 (50.4)	1740 (44.8)		
*less than once a month*	40 (11.3)	293 (13.3)	410 (13.3)	572 (14.7)		
*A few times a month*	50 (14.2)	321 (14.6)	456 (14.8)	664 (17.1)		
*A few times a week*	40 (11.3)	195 ( 8.8)	285 ( 9.2)	397 (10.2)		
*Every day and/or night*	61 (17.3)	286 (13.0)	379 (12.3)	511 (13.2)		

Data are presented as mean ± SD or n (%) ; * represent χ2 test for categorical variables; $ Linear regression models for continuous variables; UL represent urinary leakage

**Table 2:** Baseline characteristics of participants with UL

Characteristic	Urinary leakage		***P*** value
	No (*n* = 4565)	Yes (*n* = 4960)	
**Age (mean (SD)**)^$^	61.62 (10.75)	63.17 (11.02)	< 0.001
Race_2 = White (%)*	1746 (38.2)	2629 (53.0)	< 0.001
Education (%)*			0.265
*College or above*	1990 (45.0)	2232 (46.6)	
*High school or equivalent*	1859 (42.0)	1975 (41.2)	
*Less than high school*	575 (13.0)	587 (12.2)	
**Income (mean (SD))** ^$^	2.56 (1.64)	2.60 (1.59)	0.317
Drink = Yes (%)*	1373 (55.1)	1621 (61.9)	< 0.001
Smoke = Yes (%)*	1689 (37.0)	2003 (40.4)	0.001
**BMI (mean (SD))** ^$^	29.18 (7.01)	31.01 (7.59)	< 0.001
Diabetes = Yes (%)*	773 (16.9)	1025 (20.7)	< 0.001
Hypertension = Yes (%)*	2286 (50.2)	2836 (57.2)	< 0.001
Hyperlipidemia = Yes (%)*	2007 (47.3)	2518 (53.6)	< 0.001
Stroke = Yes (%)*	218 ( 4.8)	361 ( 7.3)	< 0.001
Asthma = Yes (%)*	619 (13.6)	895 (18.1)	< 0.001
Hysterectomy = Yes (%)*	1496 (33.4)	1904 (39.1)	< 0.001
Ever_pregnant_level (%)*			0.012
*1 ~ 2*	1388 (33.9)	1425 (31.3)	
*3 ~ 5*	2102 (51.4)	2479 (54.5)	
*> 5*	601 (14.7)	645 (14.2)	
Vaginal_deliveries_level (%)*			< 0.001
*0*	621 (15.1)	576 (12.6)	
*1 ~ 2*	1612 (39.2)	1797 (39.2)	
*3 ~ 5*	1552 (37.7)	1886 (41.2)	
*> 5*	332 ( 8.1)	324 ( 7.1)	
**VitD (mean (SD))** ^$^	69.61 (30.19)	72.56 (32.57)	< 0.001

Data are presented as mean ± SD or n (%) ; * represent χ2 test for categorical variables; $ Linear regression models for continuous variables; UL represent urinary leakage

**Table 3:** Baseline characteristics of participants with UL level

Characteristic	Urinary leakage					***P*** value
	L0 (*n* = 4565)	L1 (*n* = 1315)	L2 (*n* = 1491)	L3 (*n* = 917)	L4 (*n* = 1237)	
**Age (mean (SD))$**	61.62 (10.75)	60.69 (10.54)	61.69 (10.73)	63.88 (10.91)	67.05 (10.84)	< 0.001
Race_2 = White (%)*	1746 (38.2)	701 (53.3)	739 (49.6)	476 (51.9)	713 (57.6)	< 0.001
Education (%)*						< 0.001
*College or above*	1990 (45.0)	701 (54.9)	655 (45.5)	398 (45.0)	478 (40.1)	
*High school or equivalent*	1859 (42.0)	471 (36.9)	606 (42.1)	357 (40.4)	541 (45.4)	
*Less than high school*	575 (13.0)	106 ( 8.3)	179 (12.4)	129 (14.6)	173 (14.5)	
**Income (mean (SD))$**	2.56 (1.64)	3.00 (1.61)	2.62 (1.59)	2.43 (1.59)	2.25 (1.48)	< 0.001
Drink = Yes (%)*	1373 (55.1)	392 (62.0)	482 (62.0)	313 (63.5)	434 (60.8)	< 0.001
Smoke = Yes (%)*	1689 (37.0)	492 (37.4)	573 (38.5)	385 (42.0)	553 (44.7)	< 0.001
**BMI (mean (SD))$**	29.18 (7.01)	29.58 (7.12)	30.59 (7.03)	31.44 (7.26)	32.74 (8.57)	< 0.001
Diabetes = Yes (%)*	773 (16.9)	208 (15.8)	274 (18.4)	189 (20.6)	354 (28.6)	< 0.001
Hypertension = Yes (%)*	2286 (50.2)	667 (50.7)	795 (53.4)	526 (57.5)	848 (68.7)	< 0.001
Hyperlipidemia = Yes (%)*	2007 (47.3)	630 (50.7)	710 (50.2)	479 (55.4)	699 (59.2)	< 0.001
Stroke = Yes (%)*	218 ( 4.8)	61 ( 4.6)	93 ( 6.2)	63 ( 6.9)	144 (11.7)	< 0.001
Asthma = Yes (%)*	619 (13.6)	208 (15.8)	251 (16.9)	173 (18.9)	263 (21.3)	< 0.001
Hysterectomy = Yes (%)*	1496 (33.4)	435 (34.0)	536 (36.7)	361 (39.9)	572 (46.7)	< 0.001
Ever_pregnant_level (%)*						< 0.001
*1 ~ 2*	1388 (33.9)	441 (36.6)	430 (31.5)	227 (27.2)	327 (28.6)	
*3 ~ 5*	2102 (51.4)	635 (52.7)	757 (55.5)	473 (56.7)	614 (53.6)	
*> 5*	601 (14.7)	130 (10.8)	177 (13.0)	134 (16.1)	204 (17.8)	
Vaginal_deliveries_level (%)*						< 0.001
*0*	621 (15.1)	198 (16.3)	166 (12.1)	94 (11.2)	118 (10.2)	
*1 ~ 2*	1612 (39.2)	496 (40.9)	574 (41.8)	319 (37.9)	408 (35.3)	
*3 ~ 5*	1552 (37.7)	463 (38.2)	549 (40.0)	354 (42.1)	520 (45.0)	
*> 5*	332 ( 8.1)	56 ( 4.6)	84 ( 6.1)	74 ( 8.8)	110 ( 9.5)	
**VitD (mean (SD))$**	69.61 (30.19)	73.01 (32.30)	72.85 (31.57)	72.99 (34.70)	71.41 (32.44)	< 0.001

Data are presented as mean ± SD or n (%) ; * represent χ2 test for categorical variables; ^$^ Linear regression models for continuous variables; L0 represent Never; L1 represent less than once a month; L2 represent a few times a month; L3 represent a few times a week; L4 represent every day and/or night

### Vitamin D and UL

In this study, univariate and multivariate linear regression were used and constructed two main models to explore the independent impact of serum 25(OH)D on UL. As a continuous variable, serum 25(OH)D was significantly correlated with UL in both unadjusted model (odds ratio [OR]: 1.004, 95%CI: 1.003–1.004, *P* < 0.01) and adjusted model ([OR]: 1.003, 95%CI: 1.002–1.004, *P* < 0.01). When serum 25(OH)D was as a categorical variable, the relationship between serum 25(OH)D levels and UL was non-linear. Compared with serum 25(OH)D severe deficiency groups, those in the insufficient groups had 21.46% higher UL risk ([OR]: 1.2146, 95%CI: 1.006–1.460, *P* < 0.01) in the unadjusted model and 30.70% higher UL risk ([OR]: 1.307, 95%CI: 1.190–1.435, *P* < 0.01) in the adjusted model. When compared to vitamin D severe deficiency groups, the moderate deficiency groups showed an OR of 0.883 (95%CI: 0.786–0.993, *P* = 0.03) in the adjusted model (Table 4).

**Table 4:** Relationship between the serum 25(OH) D concentrations and UL in the unadjusted model and adjusted logistic regression models

	OR (95% CI) ***P*** Value	
Serum 25(OH)D concentrations	Unadjusted model ^a^	Adjusted model ^b^
**25(OH) D** *(continuous)*	1.004 (1.003, 1.004) < 0.01	1.0030 (1.002, 1.004) < 0.01
**25(OH) D** *(categorical)*		
*< 25.00*	*Reference*	*Reference*
*25.00–49.99*	0.969(0.866, 1.086) = 0.587	0.883 (0.786, 0.993) = 0.03
*50.00–74.99*	1.2146 (1.006, 1.460) < 0.01	1.307 (1.190, 1.435) < 0.01
*≥ 75.00*	0.9521 (0.999, 1.084) = 0.633	1.014 (0.949, 1.083) = 0.67

^a^ Unadjusted model represent Crude model including 25(OH) D;

^b^ Adjusted model adjusted for sociodemographic variables (including age, race, education, income and BMI), drink, smoke, diabetes, hypertension, hyperlipidemia, pregnant and vaginal deliveries history

**Fig. 2:**
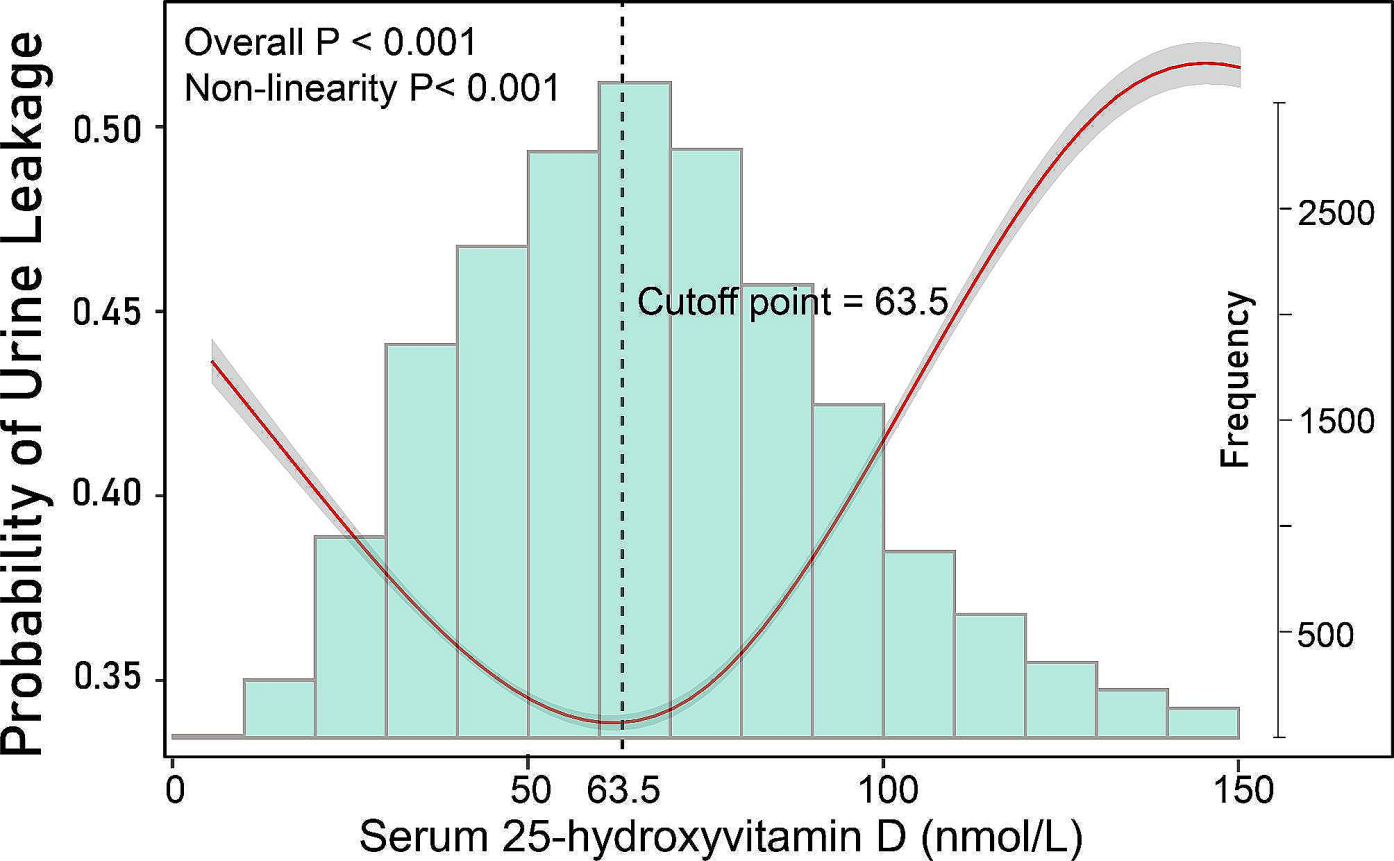
Association between serum 25(OH)D concentrations and urinary leakage (UL). Adjusted for sociodemographic variables (including age, race, education, income and BMI), drink, smoke, diabetes, hypertension, hyperlipidemia, pregnant and vaginal deliveries history. Grey area indicates the 95% confidence interval

Therefore, further analyses were carried out to investigates the relationship between serum 25(OH)D levels and UL among middle-aged and older U.S. females The adjusted smooth curve fitting revealed the association between serum 25(OH)D levels and UL was non-linear after adjusting for age, race, education, income, BMI, drink, smoke, diabetes, hypertension, hyperlipidemia, pregnant and vaginal deliveries history (Fig. 2). In order to present the relationship accurately, two-piecewise binary logistic regression was adopted. Using trial and error method, the inflection point was 63.5 nmol/L. When serum 25(OH)D concentrations above the threshold of 63.5 nmol/L, there was a positive correlation with the likelihood of UL ([OR]: 1.007, 95%CI: 1.005–1.009, *P* < 0.01). Whereas when serum 25(OH)D concentrations below 63.5 nmol/L, there was a negative correlation with the likelihood of UL ([OR]: 0.993, 95%CI: 0.989–0.996, *P* < 0.01) (Table 5).

**Table 5:** Threshold effect analysis of the relationship between the serum 25(OH) D concentrations and UL in the unadjusted model and adjusted logistic regression models

	OR (95% CI) ***P*** Value	
Serum 25(OH)D concentrations	Unadjusted model ^a^	Adjusted model ^b^
**25(OH) D < 63.5**	0.995 (0.991, 0.998) < 0.01	0.993 (0.989, 0.996) < 0.01
**25(OH) D ≥ 63.5**	1.008 (1.006, 1.010) < 0.01	1.007 (1.005, 1.009) < 0.01

^a^ Unadjusted model represent Crude model including 25(OH) D;

^b^ Adjusted model adjusted for sociodemographic variables (including age, race, education, income and BMI), drink, smoke, diabetes, hypertension, hyperlipidemia, pregnant and vaginal deliveries history

## Discussion

In this cross-sectional study, we found that serum 25(OH)D, as a continuous variable, was significantly associated with UL after adjusting for other covariates. In the categorical variables, adjusted model results suggested that serum 25(OH)D had an independent effect on UL in middle-aged and older U.S. women. Furthermore, we observed a non-linear relationship between serum 25(OH)D levels and UL, along with the potential inflection point. The risk of UL was lowest when serum 25(OH)D concentrations were approximately 63.5 nmol/L. Individuals with serum 25(OH)D levels either above or below 63.5nmol/L had an increased risk of UL. These results suggested that the independent relationship between serum 25(OH)D concentrations and UL followed an approximately U-shaped curve. As far as we are aware, this is the first study revealing the relationship between serum 25(OH)D levels and clinical UL in a large cross-sectional study of middle-aged and older U.S. women.

We found that serum 25(OH)D concentrations were associated with BMI and race. Obese individuals tend to have low levels of serum 25(OH)D because of increased storage and sequestration of serum 25(OH)D in adipose tissue [23]. The lower serum levels of 25(OH)D observed in African Americans compared to whites can be attributed to skin pigmentation, which limits vitamin D production, and the reduced intake of dietary supplements in African Americans [24, 25]. In addition, we observed that the prevalence of UL was associated with age, vaginal delivery, obesity status and education level. Some structural and functional changes that occur in the bladder with age, such as increased involuntary detrusor muscle contraction and decreased bladder elasticity and compliance, can potentially contribute to the development of UL [26]. Rortveit et al. revealed that women who deliver vaginally have a higher risk of UL [27]. Noblett et al. confirmed that obesity can place additional pressure on the pelvic floor in a state of chronic increased stress [28]. Kriss et al. [29] and Liu et al. [30] have demonstrated that individuals with lower levels of education have an elevated risk of UL, but no definitive link has been found between education and UL.

Serum 25(OH)D has a crucial role in the development of UL. It is supposed to affect UL in females in a variety of ways. An immunohistological study by Bischoff et al. suggested the presence of 1, 25-dihydroxyvitamin D3 nuclear receptors in human skeletal muscle [31]. In addition, Badalian et al. found that higher vitamin D levels have been linked to a reduced risk of pelvic floor disease in women [16]. Moreover, Parker-Autry et al. found that insufficient vitamin D was significantly associated with weakness in the levator ani muscle and coccygeus skeletal muscle, which are important components of the pelvic floor [32]. In conclusion, it is postulated that low serum 25(OH)D levels may contribute to the development of urinary incontinence by causing pelvic floor muscle weakness. This weakness, particularly in women with UL, could hinder the effective closure of the urethra during increased abdominal pressure, leading to stress urinary incontinence [33].

Another possible link between serum 25(OH)D levels and UL is inflammation. A common symptom of overactive bladder (OAB) syndrome is urge urinary incontinence (UUI). Cheung et al. found that the prevalence of UUI in patients with OAB is 82.9% [34]. Multiple studies have suggested that inflammatory cytokines play a crucial role in the regulation of connexins expression and the pathogenesis of bladder dysfunction [35, 36]. And inflammatory cytokines have been implicated in overactive parasympathetic and peptidergic/sensory interactions with local immune cells [37]. Furthermore, Zhang et al. performed a cross-sectional analysis and revealed that a pro-inflammatory diet was associated with an increased risk of UL in American women younger than 65 [38]. Calton et al. performed a systematic review and proposed that appropriate levels of serum 25 (OH) D may be essential for the optimal anti-inflammatory response of immune cells [39]. Current evidence indicated serum 25(OH)D supplements have anti-inflammatory properties [39, 40]. Consequently, we propose that low serum 25(OH)D levels may contribute to the development of urinary incontinence through inflammation.

Stafne et al. conducted a cross-sectional study of 851 healthy pregnant women and found that serum 25(OH)D levels below 50 nmol/L were associated with an increased risk of any UI, especially stress urinary incontinence (SUI) [18]. Vaughan et al. performed a prospective cohort study involving 350 community-dwelling older adults and indicated that cumulative UI events at 42 months were related to baseline serum 25(OH)D deficiency, displaying a trend towards an association [41]. In addition, a randomized controlled trial involving 60 premenopausal women demonstrated that the number of SUI and urinary leakage symptoms decreased after vitamin D supplementation in the intervention group [42]. These findings are partially consistent with our observation of a negative correlation between serum 25(OH)D levels and UL. Another interesting finding is that the clinical UL increases when serum 25(OH)D concentrations exceed the threshold. Unfortunately, there has been limited research directly exploring the connection between high levels of serum 25(OH)D and clinical UL. In addition, it is important to note that achieving serum 25(OH)D concentrations as high as 90 nmol/L through normal dietary intake or food supplements is rare or unlikely [43].

Our research presented several advantages. Firstly, compared to previous similar studies, this study derived significant benefits from a large sample size from NHANES, ensuring comprehensive documentation of serum 25(OH)D and UL data among participants. Second, given that this was an observational study, it was susceptible to potential confounders. To address this, a multi-model logistic regression analysis was employed to account for residual confounders. Third, we utilized adjusted smooth curve fitting to visualize the association between serum 25(OH)D concentration and UL and conducted threshold effect analysis.

Our study had some limitations. Firstly, the collection of UL data through questionnaire surveys introduced inevitable recall bias. Second, our cross-sectional study only established an association, but the causal relationship between serum 25(OH)D and UL remains unconfirmed. Third, the study participants were limited to 9,525 American women age 45 and above, precluding the generalization of our findings to men or individuals outside of this age range. Finally, the methods used to determine serum 25(OH)D concentration in NHANES during the 2005–2006 cycle were different from those after the 2007–2008 cycle. Although a regression equation can be used to convert serum 25(OH)D data from the 2005–2006 cycle into equivalent measurements, there may be some inherent variations in the results obtained from the two distinct measurement methods. Given these constraints, well-designed multicenter controlled trials are crucial for validating our research results.

## Conclusion

Our cross-sectional study of a representative sample of women aged 45 and above in the United States suggested a U-shaped correlation between serum 25(OH)D levels and UL. Serum 25(OH)D deficiency was found to be associated with an elevated risk of UL, independent of age and vaginal delivery. Increasing UL risk above the serum 25 (OH) D level of 63.5nmol/L remains to be further investigated. Future studies are essential to evaluate the relationship between serum 25(OH)D and UL and elucidate the underlying mechanisms of these associations.

## Data Availability

All the data are publicly available online (https://www.cdc.gov/nchs/nhanes/).
